# Editorial: Enhancing geriatric care with AI: strategies for fall prevention and aging-in-place

**DOI:** 10.3389/fragi.2026.1859857

**Published:** 2026-05-01

**Authors:** Siu Shing Man, Ronggang Zhou, Lu Peng, Alan Hoi Shou Chan

**Affiliations:** 1 School of Design, South China University of Technology, Guangzhou, China; 2 School of Economics and Management, Beihang University, Beijing, China; 3 College of Engineering, City University of Hong Kong (Dongguan), Dongguan, China; 4 College of Information Management, Nanjing Agricultural University, Nanjing, China

**Keywords:** aging-in-place, AI, fall prevention, geriatric care, strategies

The global demographic shift toward an aging population presents significant public health challenges, primarily due to the increased prevalence of age-related health issues such as balance disorders and falls. Falls are a leading cause of injury, even mortality among older adults, often resulting in severe health consequences, loss of independence, and substantial healthcare costs. This Research Topic, “Enhancing Geriatric Care with AI: Strategies for Fall Prevention and Aging-in-Place,” brings together diverse research aimed at leveraging artificial intelligence (AI) and wearable technologies to provide objective, efficient, and scalable solutions for fall risk assessment and early intervention.

Several articles in this Research Topic focus on the development and validation of advanced AI-based predictive models for fall risk. Liu et al. evaluated six machine learning algorithms to predict fall risk among community-dwelling older adults in Chinese communities. They identified the CatBoost algorithm as the most effective model, with key predictors including history of falls, comorbidities, and polypharmacy. Similarly, Yin et al. developed an XGBoost model specifically for patients with knee osteoarthritis, identifying mobility deficits, anxiety, and structural damage as synergistic high-risk factors for concerns about falling. Complementing these primary studies, Mou et al. conducted a systematic review and meta-analysis of 20 prospective studies, demonstrating that models driven by wearable device-based data, particularly those utilizing machine learning, exhibited strong discriminative ability for predicting future falls in older adults. Wu et al. further extended this risk assessment to rural settings by developing a nomogram model that integrates behavior and lifestyle factors such as whole grain consumption, frailty, and anxiety to screen high-risk rural older populations.

Advancements in sensing technology and data processing significantly promoted AI-driven strategies for fall risk assessment and intervention. Ou et al. introduced an intelligent shoe system combined with a semi-supervised learning framework that objectively predicts balance scores from plantar pressure signals, effectively mitigating the label noise inherent in clinical scales. Chen and Yao provided a comprehensive scoping review of the technological maturity gradient in this field, mapping the evolution from reactive post-fall detection to anticipatory, personalized pre-impact prediction. Their work highlights the current shift toward a “predictive-preventive-personalized” continuum in geriatric care. Legrand et al. evaluated an advanced IoT-supported platform that integrates sensor-derived gait and balance analysis with clinical assessments to detect early frailty signals in primary care settings. The results demonstrated that while sensor metrics alone have limited predictive power, a multimodal approach combining objective digital markers with clinician-rated and patient-reported data significantly improves the identification of pre-frailty and frailty.

Beyond technological development, this Research Topic also addresses the psychological and educational dimensions of fall prevention. Miao et al. developed and validated the China Health Literacy Scale for Falls in Hospitalized Older Adults, offering a specialized tool to assess a patient’s ability to understand and act on fall-related health information. Complementing this, another study by Miao et al. utilized Q-methodology to categorize hospitalized older adults into three distinct profiles, namely, fall-derivative, family-oriented, and healthy-state, based on their subjective awareness of fall risk, enabling more tailored communication by healthcare providers. Finally, Huertas-Hoyas et al. compared digital and in-person delivery of exercise programs, finding that while both reduced the fear of falling (FOF), face-to-face group exercises achieved higher adherence, emphasizing the need to optimize telematic applications for older populations. Collectively, these articles provide a multidimensional view of how AI can enhance geriatric care, from sophisticated biomechanical monitoring to personalized risk communication and community-based interventions.

